# LINC00242/miR-1-3p/*G6PD* axis regulates Warburg effect and affects gastric cancer proliferation and apoptosis

**DOI:** 10.1186/s10020-020-00259-y

**Published:** 2021-01-29

**Authors:** Peng Deng, Kai Li, Feng Gu, Tao Zhang, Wenyan Zhao, Ming Sun, Bin Hou

**Affiliations:** 1 Department of Surgical Oncology and General Surgery, Key Laboratory of Precision Diagnosis and Treatment of Gastrointestinal Tumors, Ministry of Education, The First Affiliated Hospital of China Medical University, 155 Nanjing North Street, Shenyang, 110001 Liaoning Province China; 2 Department of Ophthalmology, The First Affiliated Hospital of China Medical University, Shenyang, 110001 China; 3 Department of Stem Cells and Regenerative Medicine, China Medical University, Shenyang, 110001 China; 4 Department of General Surgery, Shengjing Hospital of China Medical University, Shenyang, 110004 China; 5 Department of Urology, Shengjing Hospital of China Medical University, Shenyang, 110004 China

**Keywords:** Aerobic glycolysis, Gastric cancer, LINC00242, miR-1-3p, G6PD

## Abstract

**Background:**

Reprogrammed glucose metabolism of enhanced Warburg effect (or aerobic glycolysis) is considered as a hallmark of cancer. Long non-coding RNAs (lncRNAs) have been certified to play a crucial role in tumor progression. The current study aims to inquire into the potential regulatory mechanism of long intergenic non-protein coding RNA 242 (LINC00242) on aerobic glycolysis in gastric cancer.

**Method:**

LINC00242, miR-1-3p and *G6PD* expression levels in gastric cancer tissues and cells were determined by qRT-PCR. Cell apoptosis or viability were examined by Flow cytometry or MTT assay. Western blot was utilized to investigate *G6PD* protein expression levels. Immunohistochemical (IHC) and hematoxylin and eosin (H&E) staining were used for histopathological detection. The targeted relationship between LINC00242 or *G6PD* and miR-1-3p was verified by luciferase reporter gene assay. Nude mouse xenograft was utilized to detect tumor formation in vivo.

**Result:**

LINC00242 and *G6PD* was high-expressed in gastric cancer tissues and cells, and LINC00242 is positively correlated with *G6PD*. Silencing of LINC00242 or *G6PD* within gastric cancer cells prominently inhibited cell proliferation and aerobic glycolysis in vitro and relieved the tumorigenesis of gastric cancer in vivo. miR-1-3p was predicted to directly target both LINC00242 and *G6PD*. Overexpression of miR-1-3p suppressed gastric cancer cells proliferation and aerobic glycolysis. LINC00242 competitively combined miR-1-3p, therefore relieving miR-1-3p-mediated suppression on *G6PD*.

**Conclusion:**

LINC00242 plays a stimulative role in gastric cancer aerobic glycolysis via regulation of miR-1-3p/ *G6PD* axis, therefore affecting gastric cancer cell proliferation.

## Introduction

Gastric cancer (GC), also recognized as stomach adenocarcinoma, is the fifth most frequent malignancies and the third leading cause of cancer-related death worldwide (Smyth et al. 2020). Gastric cancer has aroused social concern worldwide, especially in East Asian countries for the past few years (Hamashima 2014; Lordick and Terashima 2016; Sano 2017). Despite many developments had been accomplished in the aspect of surgical procedures and diagnostic approaches in surgery, chemotherapy and targeted therapy, the GC patients’ overall survival status is still remained largely unsatisfactory in recent years (Sano 2017; Yoon and Kim 2015). After radical resection of gastric cancer, nearly half of patients will suffer tumor recurrence. Distant metastases and local recurrences are the main causes of death in patients with gastric cancer (Ilson 2017). In most countries, the 5-year overall survival rate of gastric cancer patients is less than 30% (Karimi et al. 2014). The complexity of gastric cancer treatment consists in its heterogeneity within the tumor tissue, not only genetic, but also epigenetic changes (Figueiredo et al. 2017; Fu 2015). Therefore, great endeavors needed to better understand this pathological process of gastric cancer and to reveal its potential molecular mechanisms to determine potential prognostic factors and therapeutic targets.

Warburg effect (or aerobic glycolysis), a characteristic tumor cell phenotype, can expedite tumor progression by accelerating lactate production and glucose uptake (Liberti and Locasale 2016). The aerobic glycolysis not only supplies tumor cells with nutrients and ATP but also fabricates an acidic environment that results in demolition of extracellular matrix and expedites growth and metastasis (Icard et al. 2018; Lu et al. 2015). Increasing evidence suggests that the cancer-specific effects of key glycolytic enzymes can be considered as potential anti-tumor treatment strategies (Brisson et al. 2016; Ganapathy-Kanniappan and Geschwind 2013; Yi et al. 2019). In reality, the augment in activity and/or expression of some crucial glycolytic enzymes, such as lactate dehydrogenase A (LDHA)(Jin et al. 2017), hexokinase 2 (HK2) (Garcia et al. 2019) and glucose transporter isoform 1 (GLUT1) (Zambrano et al. 2019) had been discovered in a large amount of human tumors. Glucose-6-phosphate dehydrogenase (*G6PD*) is discovered in almost all cells, and is the rate-limiting step of the pentose phosphate pathway (PPP) (Stanton 2012). As a rate limiting step of PPP, *G6PD* controls how many substrates are directed to PPP and how many are allowed to enter glycolysis (Rao et al. 2015). Besides, previous researches have indicated that glycolysis is related to the malignant behavior of gastric cancer cells (Xu et al. 2018; Zhihua et al. 2019). Hence, further thorough-paced investigation on regulator modulating *G6PD* expression and Warburg effect advancement in gastric cancer might define feasible targets for gastric cancer therapy.

Noncoding RNAs are momentous constituents of the mammalian transcriptome, including long noncoding RNAs (lncRNAs, > 200 nt), microRNAs (about 20–22 nt) and so on, had already been hottest research subjects of neoplastic diseases (Bhan et al. 2017; Chan and Tay 2018). A large number of researches have manifested that lncRNAs are involved in the process of tumor through affecting gene expression via transcriptional and posttranscriptional control, chromatin remodeling or competitively binding miRNAs (Beermann et al. 2016; Dykes and Emanueli 2017). Liu et al*.* provide evidence for a novel function of the lncRNA AWPPH/miRNA-21 axis affects breast cancer cell chemosensitivity and proliferation (Liu et al. 2019a, b). Liang et al. demonstrated that lncRNA XLOC_006390 exacerbates cervical cancer metastasis and tumorigenesis as a ceRNA against miR-331-3p and miR-338-3p (Luan and Wang 2018). Considering that there are many lncRNAs and miRNAs deregulated in gastric cancer (Dan et al. 2018; Zhang et al. 2018), the research purposed to ascertain a novel lncRNA-miRNA axis regulating *G6PD* expression and Warburg effect in gastric cancer.

In the study, we analyzed datasets from Gene Expression Omnibus (GEO) to identify mRNAs regulated aerobic glycolysis in gastric cancer, and *G6PD* was selected. *G6PD* expression within gastric cancer tissue samples and cells was examined. And the biological functions of *G6PD* in gastric cancer cell proliferation, apoptosis, aerobic glycolysis progression and tumor formation were determined in vitro and in vivo. Next, miRNAs that might be correlated to *G6PD* were screened for based on bioinformatics analysis and miR-1-3p was choose. Moreover, it has been identified that miR-1-3p participated in the occurrence and development of multiple tumor diseases, like prostate cancer (Li et al. 2018), non-small-cell lung cancer (Wang et al. 2019a, b), hepatocellular carcinoma (Zhang et al. 2019) and so on. Especially, Ke et al. (Ke et al. 2019) and Chen et al. (Chen et al. 2020) suggested that miR-1-3p inhibited gastric cancer cell growth and metastasis. To further find out the upstream regulation mechanism of miR-1-3p, the supposed binding sequence between LINC00242 and miR-1-3p was predicted. The predicted miR-1-3p bindings to LINC00242 and *G6PD* were verified. The biological function of LINC00242 in gastric cancer aerobic glycolysis still unclear and need further clarification.

In the current research, the dynamic effects of LINC00242 and miR-1-3p on gastric cancer cell proliferation, apoptosis and aerobic glycolysis progression were examined. Then, the effect of knockdown of LINC00242 on the development and tumorigenesis of gastric cancer in vivo was also investigated on xenograft nude mice model. Our abundant experimental consequences furnished sufficient evidence to uncover a novel lncRNA/miRNA/mRNA axis affecting the Warburg effect progression in gastric cancer cells, proposing a momentous perception concerning the regulatory mechanism of lncRNAs in gastric cancer progression.

## Materials and methods

### Clinical tissue sampling

Human gastric cancer tissues and its corresponding adjacent non-neoplastic normal tissue specimens, which were greater than 5 cm far away from cancer edge, were retrieved from 77 gastric cancer patients diagnosed that did not undergo any chemotherapy or radiotherapy previously by pathology and cytology in the First Affiliated Hospital of China Medical University. All tissue samples were directly stockpiled in liquid nitrogen and kept at − 80 °C until use. The clinical characteristics of the gastric cancer patients are listed in Table 1. All the patients who were involved in this study consented to participate in the study and to the publication of its results. The human tissue experiments were authorized by the Ethics Committee of the First Affiliated Hospital of China Medical University.

**Table 1:** Correlation between G6PD expression and clinicopathologic characteristics in gastric cancer patients

Clinicopathologic parameters	Number	G6PD expression level		
		Low	High	*P*
Total cases	77	24	53	
Gender				0.5695
Male	38	13 (34.2%)	25 (65.8%)	
Female	39	11 (28.2%)	28 (71.8%)	
Age				0.6268
< 60	45	15 (33.3%)	30 (66.7%)	
≥ 60	32	9 (28.1%)	23 (71.9%)	
Size of tumor (cm)				0.0022*
< 5	48	21 (43.8%)	27 (56.2%)	
≥ 5	29	3 (10.3%)	26 (89.7%)	
Distant metastasis				0.0372*
M0	68	23 (33.8%)	45 (66.2%)	
M1	9	0 (0%)	9 (100%)	
TNM stage				
I + II	34	24 (70.6%)	20 (29.4%)	0.0028*
III + IV	43	10 (23.3%)	33 (76.7%)	
Perineural invasion				0.1206
Negative	56	15	42	
Positive	21	9	11	

Categorical variables were compared by the chi-square test

*P < 0.05 was recognized as a significant difference

### Cell lines and cultures

Human gastric epithelial cell line (GES-1 cell) and human gastric cancer cell lines (AGS, SGC-7901, MGC-803 and MKN-45 cells) were acquired from BeNa Culture Collection (Beijing, China). GES-1, SGC-7901, MGC-803 and MKN-45 cells were cultivated in RPMI1640 medium (Thermo Fisher Scientific, Waltham, MA, USA) supplemented with 10% fetal bovine serum (FBS) (GIBCO BRL, Grand Island, NY, USA), 100 U/ml penicillin and 100 mg/ml streptomycin; AGS cells were cultivated in F-12K medium supplemented with 10% FBS. All cells were cultured under humidified atmosphere with 5% CO_2_ at 37 ℃.

### Cell transfection

Specific short hairpin RNA (shRNA#1 and shRNA#2) of *G6PD*, shRNA of LINC00242, scramble oligonucleotides, mimics control, miR-1-3p mimics, inhibitor control and miR-1-3p inhibitor were synthesized and commercially gained from GenePharma Technology Co., Ltd (Shanghai, China). The sequences of shRNA were displayed in Table 2. AGS and MGC-803 cells were seeded in 6-well plates at a specific mass of 1 × 10^6^ per well and cultivated in incubator for 24 h at 37 °C in 5% CO_2_ until the cell confluence arrived 80–90%. Lipofectamine™ 3000 (Life Technologies, USA) was used for transfection following the instructions of manufacturer. Cells were divided into 9 groups: (i) sh-NC group (transfected with scrambled shRNA); (ii) sh-*G6PD*#1 group; (iii) sh-*G6PD*#2; (iv) sh-LINC00242 group; (v) miR-NC (transfected with blank vector of mimics; (vi) miR-1-3p mimics; (vii) sh-NC + inhibitor NC group (transfected with blank vector); (viii) miR-1-3p inhibitor; (ix) Mix group (transfected with miR-1-3p inhibitor + sh-LINC00242). After transfection, all cells were gathered for follow-up experiments.

**Table 2:** ShRNA sequence of G6PD and LINC00242

Gene	Sequence (5′–3′)
shRNA#1-G6PD	
Top strand	CACCGCAAACAGAGTGAGCCCTTCTCGAAAGAAGGGCTCACTCTGTTTGC
Bottom strand	AAAAGCAAACAGAGTGAGCCCTTCTTTCGAGAAGGGCTCACTCTGTTTGC
shRNA#2-G6PD	
Top strand	CACCGGACAACATCGCCTGCGTTATCGAAATAACGCAGGCGATGTTGTCC
Bottom strand	AAAAGGACAACATCGCCTGCGTTATTTCGATAACGCAGGCGATGTTGTCC
shRNA-NC-G6PD	
Top strand	CACCGCAAACAGAGTGAGCCCTTCTCGAAAGAAGGGCTCACTCTGTTTGC
Bottom strand	CGTTTGTCTCACTCGGGAAGAGCTTTCTTCCCGAGTGAGACAAACGAAAA
shRNA-LINC00242	
Top strand	CACCGCAGAGAATCCGAGGCTATGACGAATCATAGCCTCGGATTCTCTGC
Bottom strand	AAAAGCAGAGAATCCGAGGCTATGATTCGTCATAGCCTCGGATTCTCTGC
shRNA-NC-LINC00242	
Top strand	CACCGCAGAGAATCCGAGGCTATGACGAATCATAGCCTCGGATTCTCTGC
Bottom strand	CGTCTCTTAGGCTCCGATACTGCTTAGTATCGGAGCCTAAGAGACGAAAA

### RNA extraction and quantitative RT-PCR

Total RNA was extracted from the cells and tissues using TRIZOL™ (Invitrogen), 2.0 μg of total RNA was applied for reverse transcription using PrimeScript® Stra Strand Synthesis Kit (TaKaRa, Tokyo, Japan). Quantitative PCR was implemented by using QuantiTect® SYBR® Green RT-PCR Kit (QIAGEN, Dusseldorf, Germany). The expression level of β-actin (for lncRNA and mRNA) and U6 (for miRNA) were used as endogenous controls, respectively. The 2^−∆∆CT^ method was applied to detect the relative fold changes. The sequences of primers present in Table 3.

**Table 3:** Primer sequence for qRT-PCR

Gene	Primer	Sequence
LINC00242	forward	5′-CACTCACATCAACGGAGCCT-3′
	reverse	5′-CACTCACATCAACGGAGCCT-3′
miR-1-3p	forward	5′-ACACTCCAGGTGGGTGGAATGT-3′
	reverse	5′-CTCAACTGGTGTCGTGGAG-3′
miR-206	forward	5′-GGCGGTGGAATGTAAGGAAG-3′
	reverse	5′-GGCTGTCGTGGACTGCG-3′
miR-613	forward	5′-ACACTCCAGCTGGGAGGAATGTTCCTTC-3′
	reverse	5′-TGGTGTCGTGGAGTCG-3′
G6PD	forward	5′-AAACGGTCGTACACTTCGGG-3′
	reverse	5′-GGTAGTGGTCGATGCGGTAG-3′
β-actin	Forward	5′-CATGTACGTTGCTATCCAGGC-3′
	Reverse	5′-CTCCTTAATGTCACGCACGAT-3′
U6	Forward	5′-CTCGCTTCGGCAGCACATA-3′
	Reverse	5′-AACGATTCACGAATTTGCGT-3′

### Microarray analysis

The microarray expression data have been deposited in the Gene Expression Omnibus (GEO, https://www.ncbi.nlm.nih.gov/geo/) of the National Center of Biotechnology Information (NCBI) with accession numbers GSE51575, GSE63089 and GSE81948. The GSE51575 dataset includes 27 paired gastric carcinoma tumor tissue and normal tissues. The GSE63089 dataset includes 45 paired of gastric cancer tissues and gastric normal tissues. The GSE81948 dataset includes 15 gastric tumor tissues and 5 normal tissues. The analysis for the microarrays GSE51575, GSE63089 and GSE81948 were respectively based on the platform of GPL13607, GPL5175 and GPL6244. Aberrantly expressed genes was screened by R language analysis technique with Student’s t-test (P-value < 0.05) accompanied by |log2 (fold change)|> 1 as screening condition and exhibited by heat map.

### Western blot assay

Total protein of gastric cancer tissues or AGS and MGC-803 cells were extracted using RIPA lysate (Beyotime, Shanghai, China). After quantified by the bicinchoninic acid method (Waltham, MA, USA), 80 μg protein were segregated using SDS-polyacrylamide gel electrophoresis and transferred onto polyvinylidene difluoride (PVDF) membranes. Membranes were blocked in TBS-T buffer containing 5% nonfat milk for 1 h. Then the membranes experienced incubation with primary antibodies including anti-*G6PD* antibody (Cat# ab133525, 1/1000, Abcam, Cambridge, MA, USA) at 4 °C overnight. After washed using TBST thrice, the membranes were then hybridized with the horseradish peroxidase (HRP)-linked secondary antibody rabbit anti-mouse IgG H&L (Cat# ab6728, 1/2000, Abcam) at room temperature for 1.5 h. Signal detection was carried out with an ECL system (Life technologies corporation, Gaithersburg, MD, USA), and β-actin was detected as control groups.

### Cell counting kit-8 (CCK-8)

CCK-8 kit (Dojindo, Kumamoto, Japan) was applied to detect the influence of LINC00242, miR-1-3p or *G6PD* on cell proliferation. Briefly, AGS and MGC-803 cells with concentration of 2 × 10^3^ per well were plated in the 96-well plate and cultivated for 0 h, 24 h, 48 h, 72 h, respectively. At the specified time, 10 µl CCK-8 was append ed and the cells were cultivated for another 4 h. SpectraMax M5 microplate reader (Molecular Devices, Sunnyvale, CA, USA) was used to determine under the experiment condition of 450 nm. All procedures were repeated at least three times.

### Flow cytometry for cell apoptosis

Flow cytometry was used to detect apoptosis. Collect cells after digestion by trypsin and resuspend the cells with 100 μl of binding buffer. Then, add 5 μl of Annexin V-FITC and 5 μl of Propidium Iodide (PI) and incubate the cells at 25℃ in the dark for 20 min. At the end of the incubation, cell apoptosis was examined on flow cytometry.

### Glycolysis analysis

Lactate production and glucose consumption were measured by the Lactate Colorimetric Assay Kit (Merck Millipore, MA, USA)) and Glucose Uptake Colorimetric Assay Kit (Sigma-Aldrich, MO, USA), respectively, according to the manufacturer's protocols and previously described (Mao et al. 2019). RT-PCR was applied to detect the glycolytic enzymes expression levels. All procedures were repeated at least three times.

### Luciferase assay

The targeted correlation between LINC00242 and miR-1-3p was predicted using ENCORI database (http://starbase.sysu.edu.cn). Then, targeted relationship between miR-1-3p and *G6PD* was predicted using TargetScan v7.2 database (http://www.targetscan.org/vert_72/). PmirGLO, pmirGLO-LINC00242-wt or pmirGLO-LINC00242-mut, pmirGLO-*G6PD*-wt or pmirGLO-*G6PD*-mut were commercially acquired from Youbio (Changsha, China), and then was co-transfected with miR-1-3p mimics/inhibitor or mimics/inhibitor control into 293 T cells by Lipofectamine-mediated gene transfer. Cells were collected 48 h after transfection and luciferase activity was determined according to the protocol of dual-luciferase reporting assay system (Promega, Madison, WI, USA).

### Construction of shRNA lentiviral vectors

A unit of 20 μg of *G6PD* and LINC00242 shRNA or scrambled shRNA was established into BLOCK-iT™ Lentiviral RNAi expression system (Invitrogen), which were applied to restrain the generation of *G6PD* and LINC00242 in AGS cells. Briefly, AGS cells with a concentration of 3 × 10^5^ cells per dish were plated into 35 mm dishes. After 1 day of cultivation for cells, 200 μl of lentiviral particles in 2 mL RPMI-1640 medium containing 10% FBS were appended into the cultures, then cells were cultivated for a day in 5% CO_2_ at 37 °C. The cells which were successfully transfected by lentiviral particles (Lv-sh-NC or Lv-sh-*G6PD*#1*,* Lv-sh-*G6PD*#2, and Lv-sh-LINC00242) were chosen using 3 μg/ml puromycin supplied with 10% FBS for 48 h. The cells were then harvested and subjected to qRT-PCR assay to verify the transfection efficiency of used lentiviral particles for restraining *G6PD* and LINC00242 gene expression.

### Xenograft mice assay in vivo

Pathogen free conditions were kept through the lifetime of 30 male BALB/c nude mice (4 weeks old). The trial license of xenograft in vivo assay was acquired from the First Affiliated Hospital of China Medical University. Firstly, the serum free cell suspensions of untreated AGS cells (1 × 10^6^) were injected subcutaneously into the back of nude mice, then nude mice were fed until the 7st day. Subsequently Lentiviral-sh-AC005224.4 or *G6PD*#1 and *G6PD*#2 transfected AGS cells (1 × 10^6^) or Lentiviral-sh-NC transfected AGS cells were transfused into the tumor of nude mice. Caliper was employed to detect tumor volume according to the formula of length × width^2/2^. The average volume of tumor was scaled for 3 times every 3 days. At the termination of the experiment (the 25th day), mice were sacrificed and the tumor was fetched from each mouse to determine the average weight and volume.

### Histopathology examination

The histopathology examination of gastric cancer tissues was performed using hematoxylin eosin (HE) staining. The gastric cancer tissues samples were put in 10% formaldehyde solution, dehydrated in ethanol gradient, embedded in paraffin, and cut down into slices of 4 μm. After deparaffinage, the samples were stained using hematoxylin and eosin. Then the slices were mounted and observed under a light microscope (Leica Microsystems, Wetzlar, Germany).

### Immunohistochemistry (IHC)

Immunohistochemical staining was applied on 4 μm-thick slices of mice tumor tissues. In short, the tissues were embedded in paraffin, then the slices were deparaffinized and rehydrated through graded alcohols and washed in Phosphate Buffered Saline (PBS) for 2 times with 10 min. The next sections were incubated the slices overnight with rabbit polyclonal primary antibody of *G6PD* (Cat# ab133525, Abcam). Then the sections were incubated with 45 µl secondary antibody horseradish peroxidase-conjugated goat polyclonal anti-mouse IgG H&L (HRP) (1:500, ab6789, Abcam) at 37℃ for 30 min. Slices were stained with 3,3′-diaminobenzidine (DAB) working solution for 3 min, then washed in water for 10 min. Slices were counterstained with hematoxylin. After rewashing the slices in water for 10 min, we finally dehydrated and cleared. The slices were ready at the time for microscopic observation.

### Statistical analysis

The animal experiments were implemented at least six independent times. The cell experiments were implemented for three independent times at lowest. The data were emerged as the mean ± SD. Statistical calculation were implemented with SPSS20.0 statistical software. Student's t-test was applied to contrast the differences between two experimental groups. One-way ANOVA was applied to analyze three groups or above. P < 0.05 was deemed as a statistically significant difference.

## Results

### Expression and prognosis significance of *G6PD* in gastric cancer

**Fig. 1:**
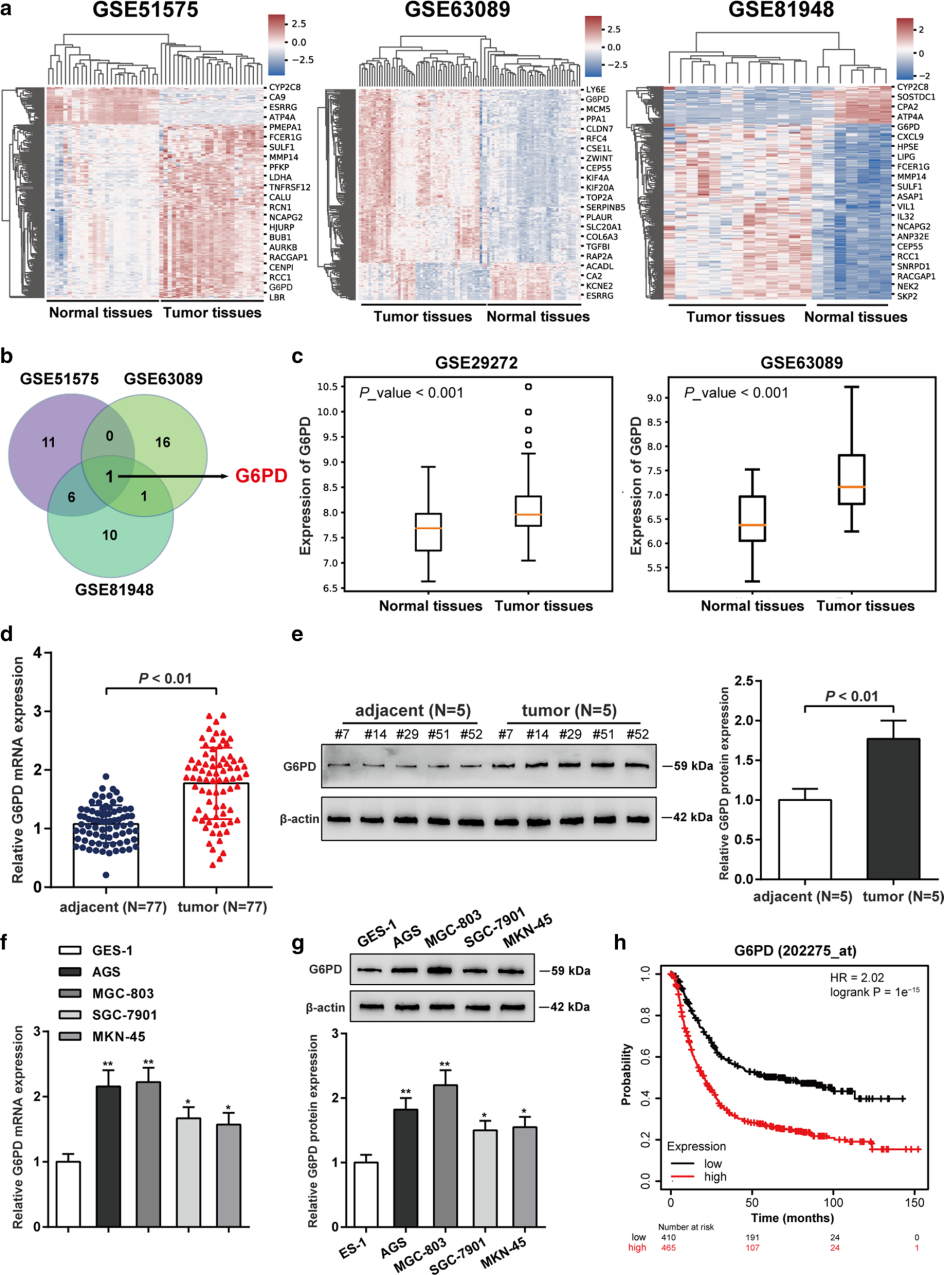
mRNA microarray analysis and up-regulated expression of *G6PD* in gastric cancer. **a** The heat map illustrated the differently expressed mRNAs in gastric tumor and normal tissues based on online datasets GSE51575, GSE63089 and GSE81948. **b** *G6PD* was both highly expressed in gastric cancer tissue samples of GSE51575, GSE63089 and GSE81948 datasets. **c** The expression levels of *G6PD* in gastric cancer tissue samples and corresponding adjacent normal tissue samples, based on online datasets GSE29272 and GSE63089. **d** The mRNA expression of *G6PD* was examined in 77 paired gastric cancer and adjacent non-cancerous tissues by qRT-PCR. **e** The protein levels of *G6PD* were examined in tissue samples by western blot. **f** The mRNA expression of *G6PD* was detected in human gastric epithelial cell line (GES-1) and gastric cancer cell lines (AGS, MGC-803, SGC-7901 and MKN-45 cells) by qRT-PCR. **g** The protein levels of *G6PD* was examined i human gastric epithelial cell line and gastric cancer cell line by western blot. ^*^P < 0.05, ^**^P < 0.01 compared to GES-1 group. **h** Kaplan–Meier survival analysis confirmed that the low expression of *G6PD* was related to the better prognosis of gastric cancer patients (log-rank test, *P* < 0.01)

Based on microarray analyses, we respectively filtered out 18 highly expressed mRNAs and 4 lowly expressed mRNAs in the gastric cancer tissue samples based on GEO microarray (accession numbers: GSE51575, GSE63089 and GSE81948) and visualized them in heat-maps (Fig. 1a). There is only one mRNA (*G6PD*) both highly expressed in gastric cancer tissue samples of GSE51575, GSE63089 and GSE81948 microarray (Fig. 1b). As we have mentioned, Glucose-6-phosphate dehydrogenase (*G6PD*) is a rate-limiting enzyme of the pentose phosphate pathway (Chen et al. 2018; Yang et al. 2019) and a tumor promoter in numerous tumor types (Yang et al. 2018; Zhang et al. 2019). Then, this study investigated the *G6PD* expression levels was observably up-regulated in gastric cancer tissue samples based on online datasets GSE29272 and GSE63089 (Fig. 1c). The *G6PD* mRNA expression level in 77 gastric cancer tissue samples was dramatically increased when compared with that corresponding adjacent normal tissue samples (*P* < 0.01, Fig. 1d). Consistently, the protein level of *G6PD* was significantly up-regulated in gastric tumor tissues compared with those in adjacent non-cancerous tissues (*P* < 0.01, Fig. 1e). The correlation between gastric cancer patients’ clinicopathological features and *G6PD* expression was displayed in Table 1. There was no remarkable relation between *G6PD* level and gender, age or perineural invasion, while patients with high expression of *G6PD* are more feasible to shows a higher distant metastasis and TNM staging (*P* < 0.05). In cell lines, *G6PD* expression mRNA and protein levels were dramatically high-expressed in different gastric cancer cell line (AGS, MGC-803, SGC-7901 and MKN-45 cells) compared with human gastric epithelial cell line (GES-1) and found that *G6PD* expression was higher in MGC-803 and AGS cells in four cancer cells (*P* < 0.01, Fig. 1f, g). So, we selected AGS and MGC-803 cells as our experimental objects in the follow-up experiments. In addition, the overall survival analysis of patients with high-expressed *G6PD* was significantly poorer than those with low-expressed *G6PD* (*P* < 0.01, Fig. 1h). All these results proved that overexpressed *G6PD* was observed in gastric cancer tissues and cells, which may in correlation with gastric cancer patient’s poor survival rate.

### Effects of *G6PD* silencing on glycolysis in gastric cancer cells

**Fig. 2:**
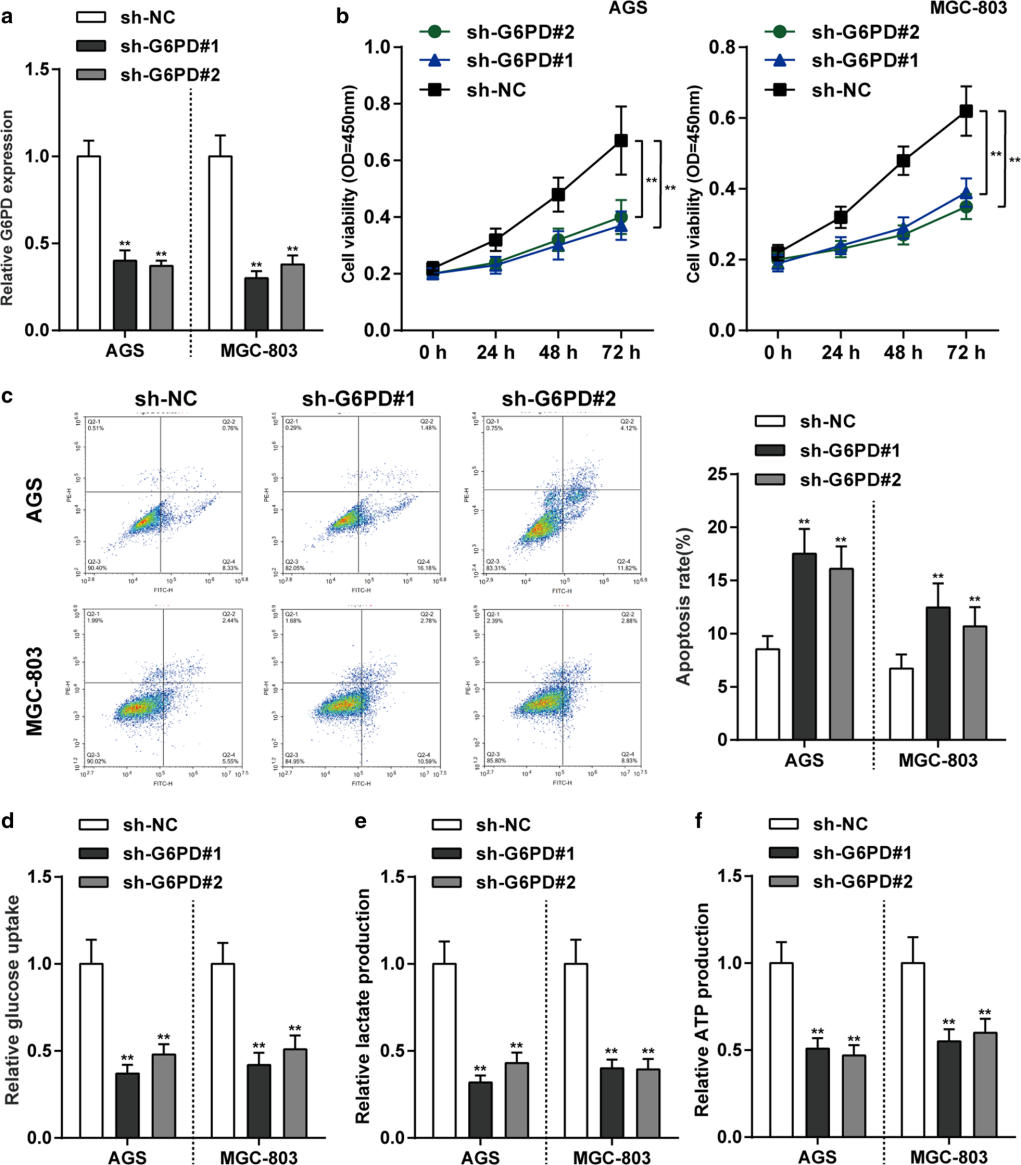
Effects of *G6PD* silencing on glycolysis in gastric cancer cells. **a** *G6PD* knockdown was achieved in AGS and MGC-803 cells by transfecting short hairpin RNA targeting *G6PD* (sh-*G6PD*#1 and sh-*G6PD*#2). The *G6PD* mRNA expression level was confirmed by qRT-PCR. **b, c** AGS and MGC-803 cells were transfected with sh-*G6PD*#1 and sh-*G6PD*#2 and examined for cell viability by MTT assay (**b**) and cell apoptosis by Flow Cytometry assay **c**. **d**–**f** Knockdown of *G6PD* expression significantly decreased glucose consumption (**d**), lactate production (**e**) and ATP production (**f**). N = 3, **P < 0.01 compared to sh-NC group

The abnormal high-expression of *G6PD* suggests that it may serve as a tumor promoter against gastric cancer. For verifying the speculation, we achieved *G6PD* silencing in AGS and MGC-803 cells by transfecting short hairpin RNA targeting *G6PD* (sh-*G6PD*#1 and sh-*G6PD*#2). The *G6PD* expression level was confirmed by qRT-PCR and *G6PD* knockdown was successfully conducted (*P* < 0.01, Fig. 2a). CCK-8 assay showed that cell proliferation ability in both MGC-803 and AGS cells were significantly inhibited by knockdown of *G6PD* (*P* < 0.01, Fig. 2b). Then, knockdown of *G6PD* observably facilitated cell apoptosis in both MGC-803 and AGS cells (*P* < 0.01, Fig. 2c). As for the glycolysis progression, silent of *G6PD* expression notably reduced the glucose utilization, lactate concentrations, and ATP production in both AGS and MGC-803 cells (*P* < 0.01, Fig. 2d–f). These data indicated that knockdown of *G6PD* inhibits gastric cancer cell proliferation and glycolysis.

### Knockdown of *G6PD* suppressed gastric cancer cell growth in vivo

**Fig. 3:**
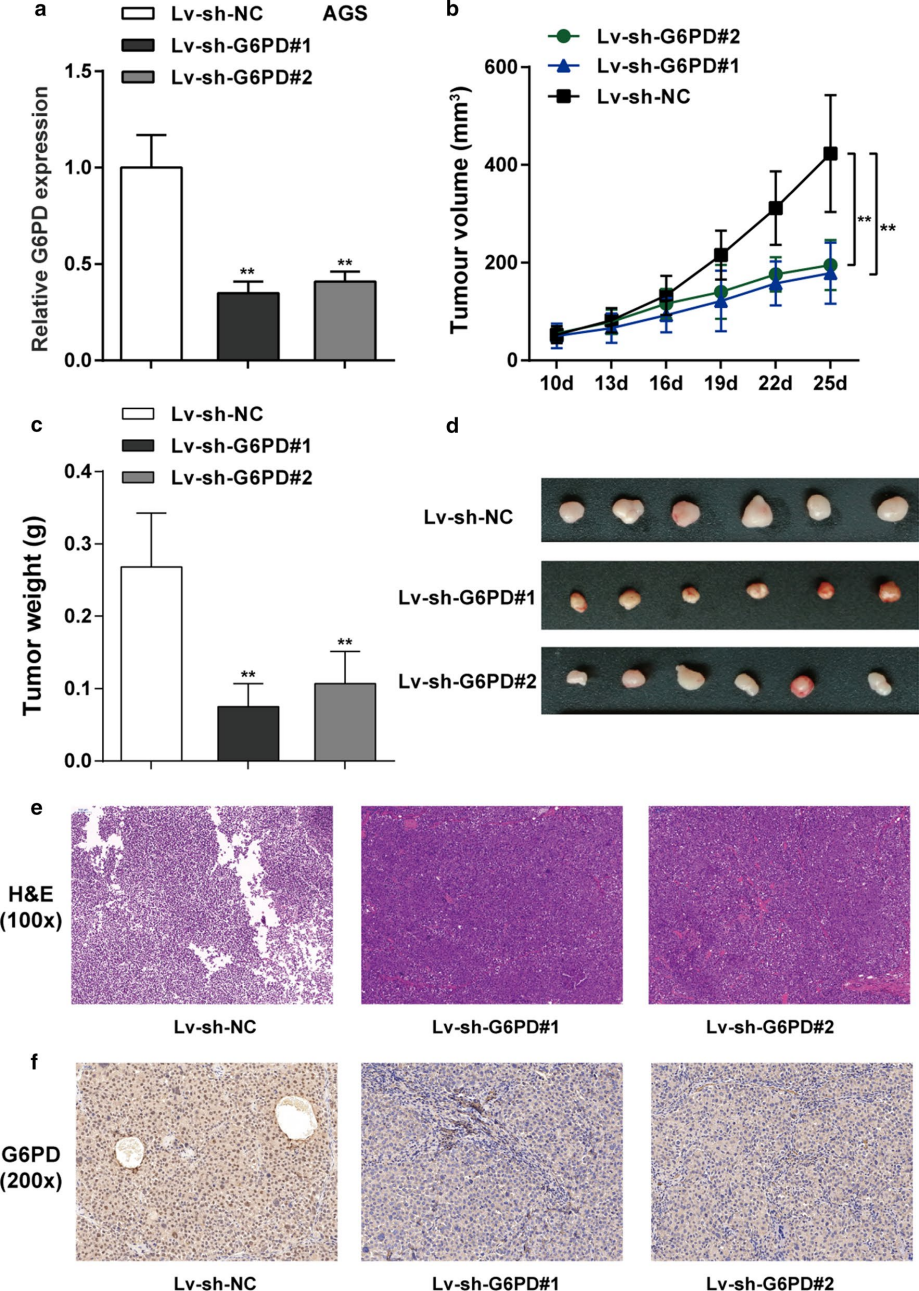
Knockdown of *G6PD* inhibited tumor formation in nude mice. **a** Subcutaneous xenotransplant tumor model was established in nude mice by injecting AGS cells infected with lentivirus-mediated shRNA targeting *G6PD* (sh-*G6PD*#1 and sh-*G6PD*#2) or negative control. The *G6PD* mRNA expression level in tumor tissues was examined by real-time qPCR. **b** Tumor volume was measured every three days from the tenth day after injection, when the tumor began to form. **c** On the 25th day, nude mice were sacrificed and the tumor weight was measured. **d** Images of the tumors in each group. **e** The histopathological characteristics of the tumors were examined by hematoxylin and eosin (H&E) staining (100 ×). **f** The protein content and distribution of *G6PD* in tissue samples were examined using Immunohistochemical (IHC) staining (× 200). N = 6; **P < 0.01, compared with Lv-sh-NC group

Mouse tumor xenograft model was employed to investigate the impacts of *G6PD* on gastric cancer in vivo. AGS cells co-transfected with lentivirus-mediated sh-*G6PD*#1 and sh-*G6PD*#2 or shRNA-control were subcutaneous injected into the back of nude mice respectively. The results of PCR assay indicated that *G6PD* expression level was significantly down-regulated in sh-*G6PD*#1 and sh-*G6PD*#2 group compared with negative control group, indicating successful transfection (*P* < 0.01, Fig. 3a). At the 7th day of the experiment, the tumor volume was determined every three days, and the AGS cells with silence of *G6PD* visibly generated smaller tumor compared with that tumor in the sh-NC group (*P* < 0.01, Fig. 3b). At the destination of the experiment (the 25th day), mice were painlessly killed and the tumor was taken out. The tumor weight in sh-*G6PD* group was notably restrained compared to sh-NC group (Fig. 3c, d). Moreover, the results of H&E staining in gastric cancer tissues demonstrated that tumor tissues in sh-NC group showed obvious necrosis, whereas silencing of *G6PD* improved the necrosis in tumor (Fig. 3e). As displayed by IHC staining, the brown color demonstrates the *G6PD*-positive IHC staining and the staining density and staining intensity were lower in gastric cancer tissues of sh-*G6PD* group than that in normal gastric cancer tissues (Fig. 3f). These data suggested that knockdown of *G6PD* efficiently relieved the tumorigenesis of gastric cancer in vivo.

### Target relationship between *G6PD* and miR-1-3p

**Fig. 4:**
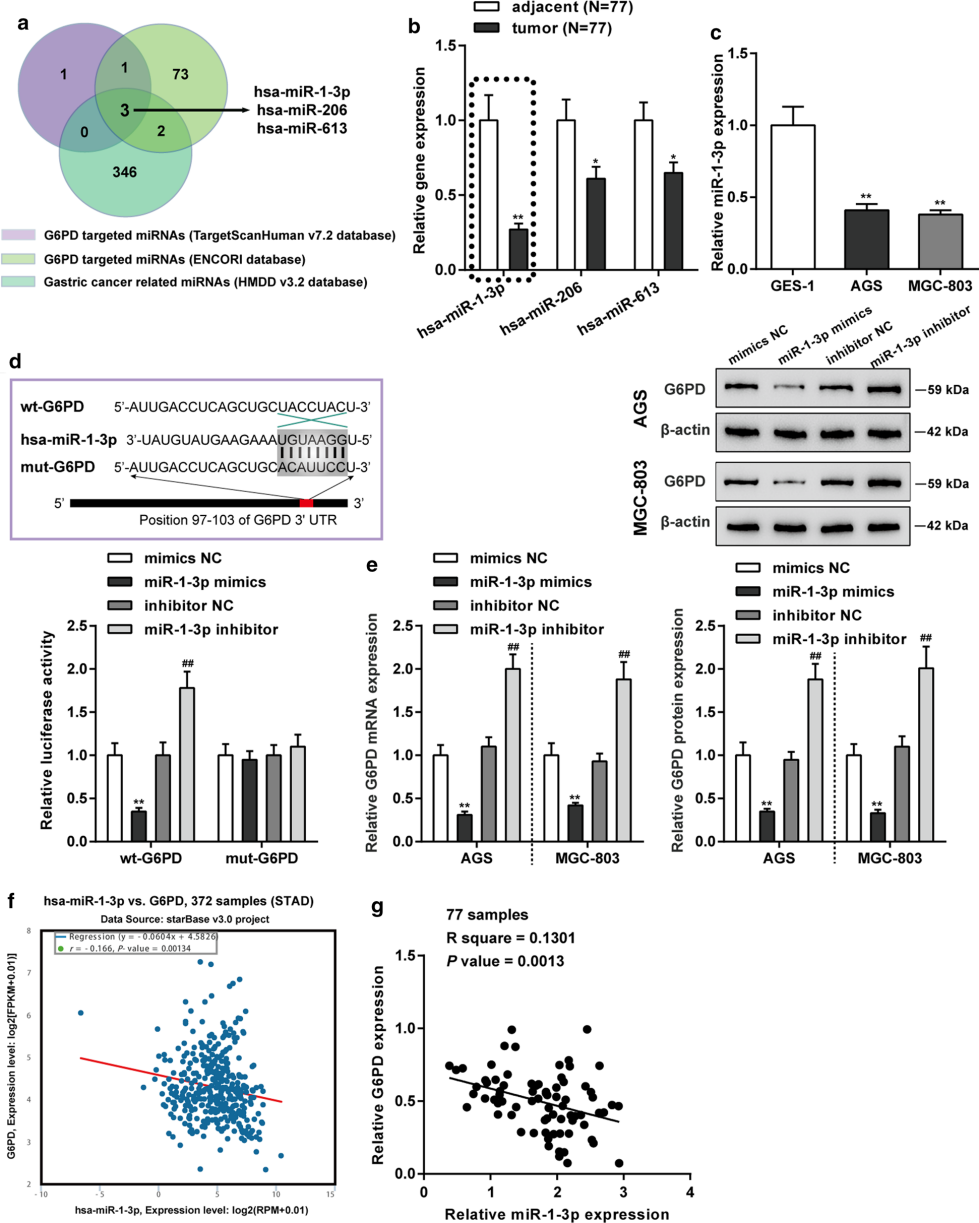
Targeting relationship was identified between *G6PD* and miR-1-3p. **a** Three miRNAs (hsa-miR-1-3p, hsa-miR-206 and hsa-miR-613) targeted *G6PD* in gastric cancer was screened according ENCORI, TargetScan v7.2 and HMDD v3.2 databases. **b** RT-PCR assay was performed to detect the expression level of these three miRNAs in gastric cancer tissues and paracancerous tissues. N = 77; *P < 0.05, **P < 0.01 compared to adjacent tissues**. c** The miR-1-3p expression level in gastric cancer cell lines (AGS and MGC-803) and gastric epithelial cell line (GES-1). N = 3; *P < 0.01 compared to GES-1 cell. **d** Prediction of binding sites of miR-1-3p and *G6PD*. And the targeting relationship between miR-1-3p and *G6PD* was verified by luciferase reporter assay in 293 T cell. N = 3; **P < 0.01 compared to mimics NC group; ^##^*P* < 0.01 compared to inhibitor NC group. **e** The effect of miR-1-3p on *G6PD* mRNA and protein expression in AGS and MGC-803 cells was verified by qRT-PCR and western blot assay. N = 3; **P < 0.01 compared to mimics NC group; ^##^*P* < 0.01 compared to inhibitor NC group. **f** A negative correlation between miR-1-3p and *G6PD* was verified based on ENCORI databases. **g** The linear analysis revealed a negative correlation between miR-1-3p and *G6PD* expression in gastric cancer tissues (N = 77)

To explore miRNAs which regulates *G6PD* in gastric cancer, bioinformatics analysis (ENCORI database and TargetScan V7.2 database) was used to predict targeted miRNAs for *G6PD*. In the meantime, the Human microRNA Disease Database (HMDD v3.2) was applied to predict miRNAs related to the progress of gastric cancer. There are three miRNAs (hsa-miR-1-3p, hsa-miR-613 and hsa-miR-206) targeted *G6PD* in gastric cancer (Fig. 4a). Among them, miR-1-3p showed most marked decrease in gastric cancer patient tissue samples compared that in normal tissue samples (*P* < 0.05, Fig. 4b). The miR-1-3p expression level in gastric cancer cell lines (AGS and MGC-803) were significantly lower than gastric epithelial cell line (GES-1) (*P* < 0.01, Fig. 4c). Underlying binding sites between miR-1-3p and *G6PD* was shown in Fig. 4d. Then, the targeted relationships between miR-1-3p and *G6PD* were validated by luciferase activity assay (Fig. 4d). As showed in Fig. 4e, overexpression of miR-140-3p observably inhibited *G6PD* mRNA and protein expression level, whereas miR-140-3p inhibitor markedly motivated *G6PD* mRNA and protein expression level in both MGC-803 and AGS cells (*P* < 0.01). Based on ENCORI database, the expression levels of miR-1-3p and *G6PD* was observably negatively correlated in gastric cancer (R = − 0.166, *P* = 0.00134, Fig. 4f). Linear correlation was adopted to ascertain the relationship between miR-1-3p expression and *G6PD* expression in 77 gastric cancer tissue samples. A negative correlation between miR-1-3p and *G6PD* was confirmed as the R = − 0.3607 and *P* = 0.0013 (Fig. 4g). These consequences disclosed that miR-1-3p targeted *G6PD* and regulated *G6PD* expression in gastric cancer.

### Effects of miR-1-3p on glycolysis within gastric cancer cells

**Fig. 5:**
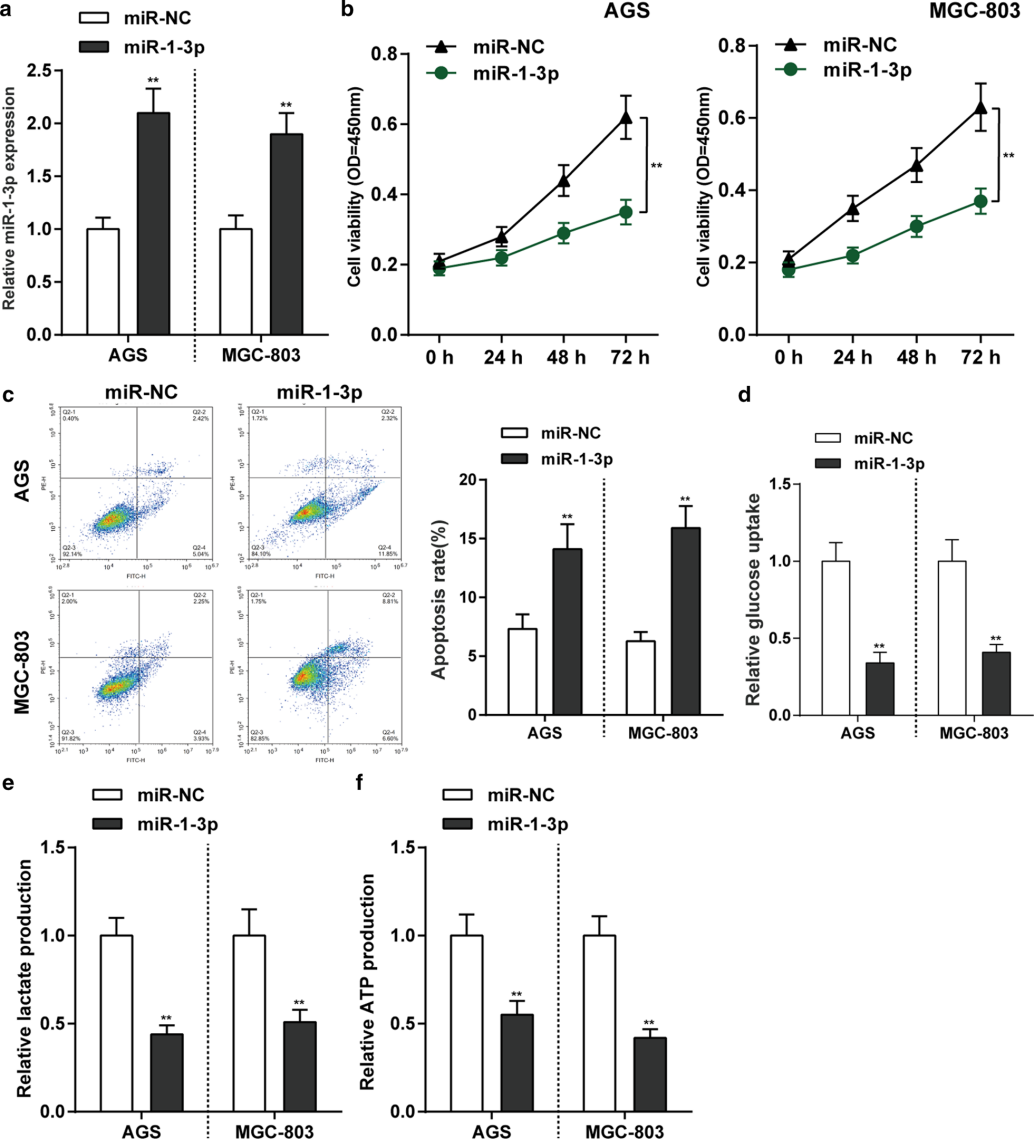
Effects of miR-1-3p on glycolysis in gastric cancer cells. **a** miR-1-3p overexpression was conducted in AGS and MGC-803 cells by transfecting miR-1-3p mimics. The transfection efficiency was verified using real-time PCR. **b** Cell viability was detected by MTT assay. **c** Cell apoptosis was determined by Flow Cytometry assay. **d–f** Overexpression of miR-1-3p markedly inhibited glucose consumption (**d**), lactate production (**e**) and ATP production (**f**) in both AGS and MGC-803 cells. N = 3; **P < 0.01 compared to miR-NC group

The study firstly conducted miR-1-3p overexpression in AGS and MGC-803 cells by transfecting miR-1-3p mimics, as validate d by PCR analysis (Fig. 5a). Overexpression of miR-1-3p dramatically reduced the viability (*P* < 0.01, Fig. 5b) while increased the apoptosis of both MGC-803 and AGS cells (Fig. 4g). As for the glycolysis progression, as demonstrated in Fig. 5d–f, overexpression of miR-1-3p memorably inhibited glucose consumption (Fig. 5d), lactate production (Fig. 5e), and ATP production (Fig. 5f) in both MGC-803 and AGS cells. These outcomes concluded that overexpression of miR-1-3p restrained gastric cancer cell proliferation and glycolysis, possibly in a *G6PD*-related way.

### LINC00242 was up-regulated in gastric cancer tissues and cell

**Fig. 6:**
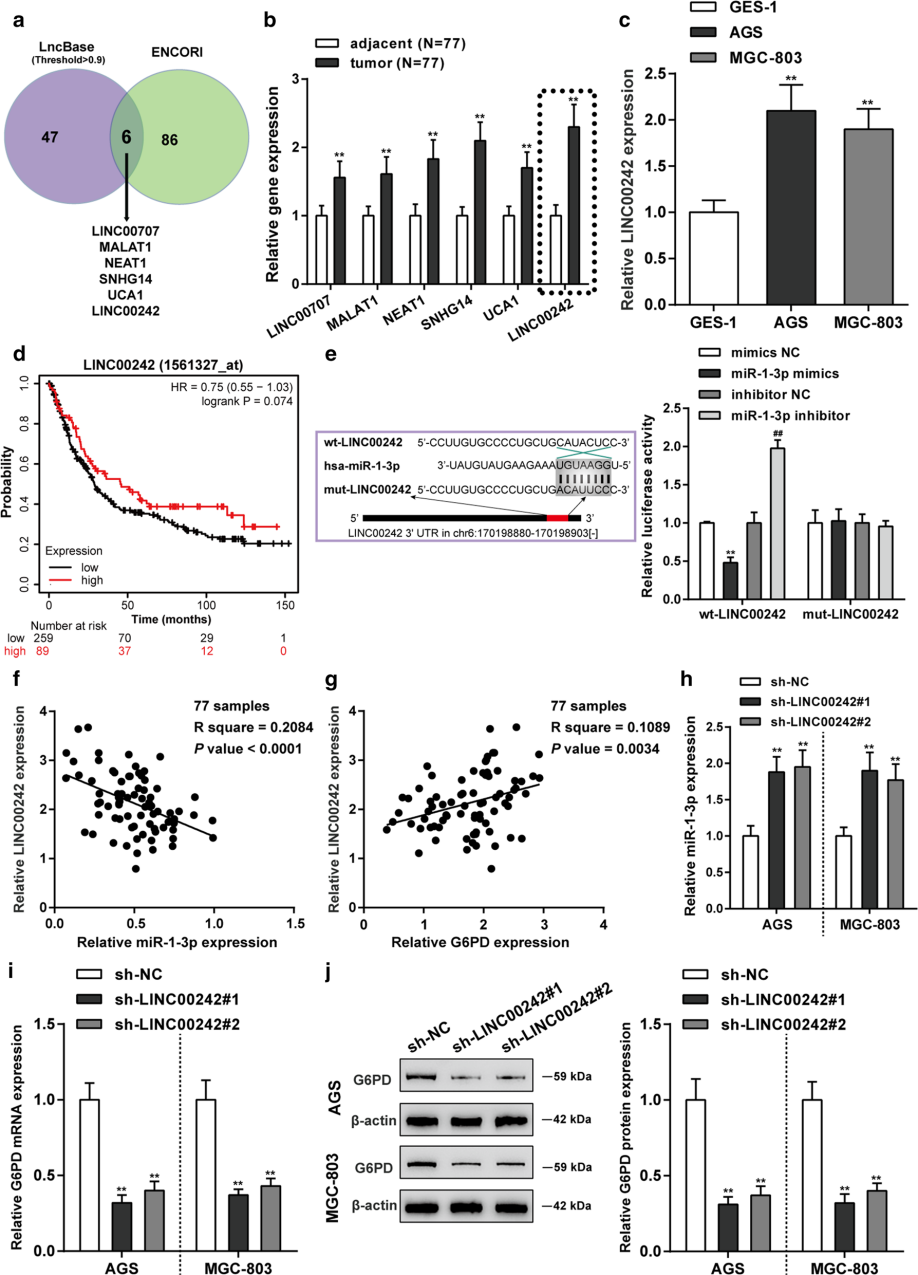
The regulatory network of LINC00242/ miR-1-3p/ *G6PD* axis. **a** Six lncRNAs (LINC00707, MALAT1, NEAT1, SNHG14, UCA1 and LINC00242) targeted miR-1-3p was screened according LncBase Predicted v.2 database and ENCORI database. **b** RT-PCR assay was performed to detect the expression level of these six lncRNAs in gastric cancer tissues and paracancerous tissues. N = 77; **P < 0.01 compared to adjacent tissues.** c** LINC00242 expression level in gastric cancer cell lines (AGS and MGC-803) and gastric epithelial cell line (GES-1) was detected. N = 3; *P < 0.01 compared to GES-1 cell. **d** Kaplan–Meier survival analysis confirmed that the low expression of LINC00242 was related to the better prognosis of gastric cancer patients (log-rank test, *P* < 0.05). **e** Target relationship between LINC00242 and miR-1-3p was predicted by bioinformatics analysis (left) and validated by dual-luciferase reporter assay (right). N = 3; **P < 0.01 compared to mimics NC group; ^##^*P* < 0.01 compared to inhibitor NC group. **f** The linear analysis revealed a negative correlation between LINC00242 and miR-1-3p expression in gastric cancer tissues (N = 77). **g** The linear analysis revealed a positive correlation between LINC00242 and *G6PD* expression in gastric cancer tissues (N = 77). **h** The effect of knockdown of LINC00242 on miR-1-3p expression in AGS and MGC-803 cells was verified by qRT-PCR. N = 3; **P < 0.01 compared to sh-NC group. **i**, **j** The effect of silencing of LINC00242 on *G6PD* mRNA and protein expression in AGS and MGC-803 cells was verified by qRT-PCR and western blot assay. N = 3; **P < 0.01 compared to sh-NC group

To further find out the upstream regulation mechanism of miR-1-3p, bioinformatics analysis (LncBase Predicted v.2 database and ENCORI database) was used to predict targeted lncRNAs for miR-1-3p. There are six lncRNAs (LINC00707, MALAT1, NEAT1, SNHG14, UCA1 and LINC00242) targeted miR-1-3p (Fig. 6a). Among them, LINC00242 emerged the most marked increase in gastric cancer patient tissue samples compared that in normal tissue samples (*P* < 0.01, Fig. 6b). And LINC00242 expression level in gastric cancer cell lines (AGS and MGC-803) were prominently higher than gastric epithelial cell line (GES-1) (*P* < 0.01, Fig. 6c). Therefore, LINC00242 was selected as the research object. The overall survival analysis of patients with high-expressed LINC00242 was notably poorer than those with low-expressed LINC00242 (*P* < 0.05, Fig. 6d). The putative binding sequence between LINC00242 and miR-1-3p is predicted according the online database ENCORI. Then luciferase activity assay verified the targeting binding relationship between LINC00242 and miR-1-3p (Fig. 6e). LINC00242 exhibited a negative correlation with miR-1-3p in 77 gastric cancer tissues (R = − 0.4565, *P* < 0.0001, Fig. 6f); LINC00242 and *G6PD* appeared a positive relevance with each other in 77 gastric cancer tissues (R = 0.3300, *P* = 0.0034, Fig. 6g). As displayed in Fig. 6h, silence of LINC00242 significantly facilitated miR-1-3p expression level in both MGC-803 and AGS cells (*P* < 0.01). On the contrary, knockdown of LINC00242 dramatically inhibited *G6PD* mRNA (Fig. 6i) and protein (Fig. 6j) expression level (*P* < 0.01). These results disclosed that LINC00242 was high-expressed in both gastric cancer tissues and cells and LINC00242/miR-1-3p/*G6PD* regulatory axis may contributes to the tumorigenesis of gastric cancer.

### Dynamic effects of LINC00242 and miR-1-3p on glycolysis within gastric cancer cells

**Fig. 7:**
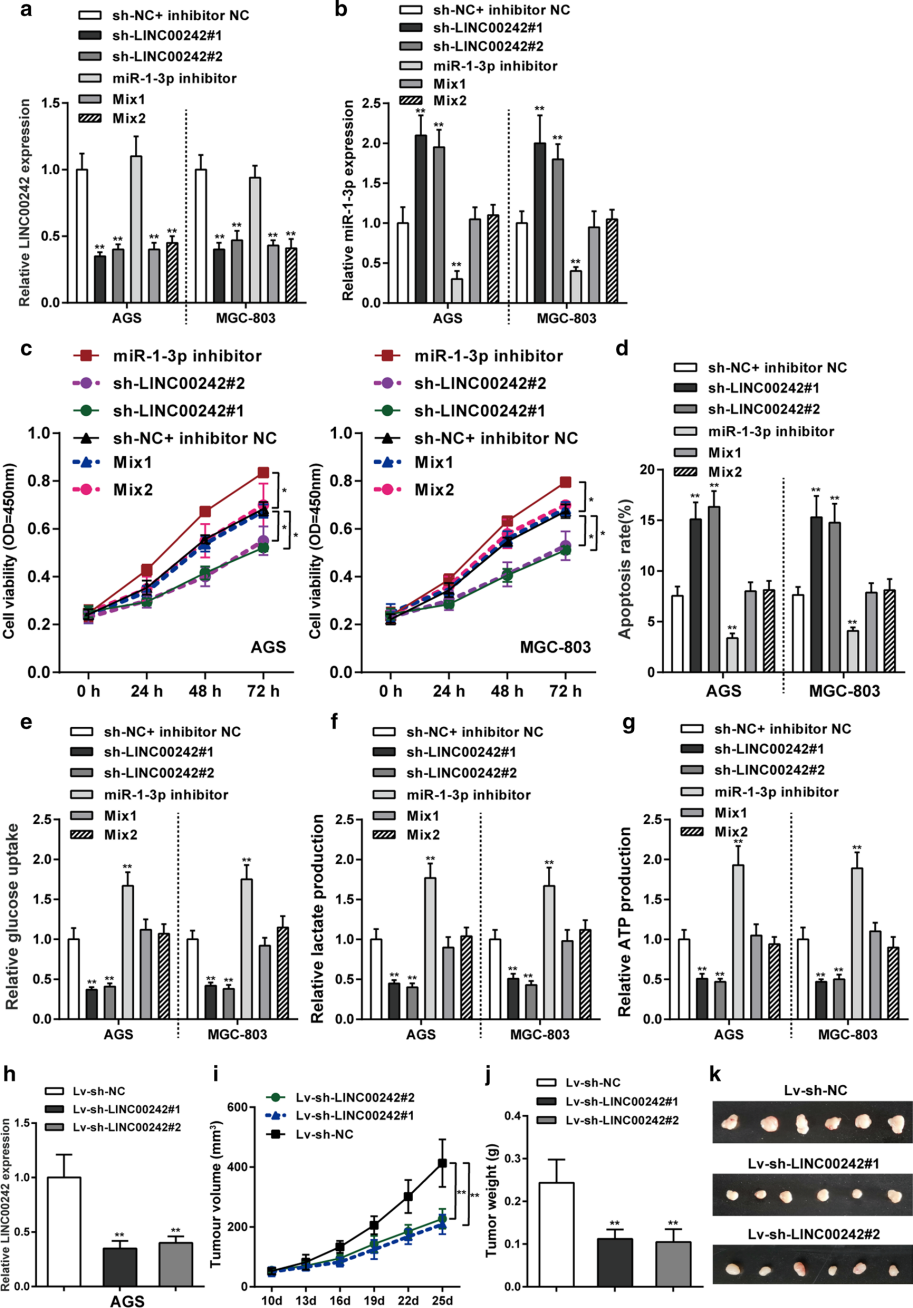
Effects of LINC00242 knockdown on glycolysis and growth of gastric cancer cells in vitro and in vivo. **a** AGS and MGC-803 cells were separated into six groups: sh-NC + inhibitor NC group (cells transfected with empty plasmid), sh-LINC00242#1 group (cells transfected with shRNA-LINC00242#1), sh-LINC00242#2 group (cells transfected with shRNA-LINC00242#2), miR-1-3p inhibitor group (cells transfected with miR-1-3p inhibitor), Mix1 group (cells transfected with shRNA-LINC00242#1 + miR-1-3p inhibitor) and Mix2 group (cells transfected with shRNA-LINC00242#2 + miR-1-3p inhibitor).. The LINC00242 mRNA expression level was detected in four experiment groups by qRT-PCR. **b** The miR-1-3p mRNA expression level was detected in four experiment groups by qRT-PCR. **c** Cell viability was detected by MTT assay. **d** Cell apoptosis was determined by Flow Cytometry assay. **e–g** Glucose consumption (**e**), lactate production (**f**) and ATP production (**g**) were determined in four experiment groups. N = 3; **P < 0.01 compared to sh-NC + inhibitor NC group. **h** AGS cells pre-transfected with lentivirus-mediated shRNA-LINC00242#1, shRNA-LINC00242#2 or shRNA-control were subcutaneous injected into the back of nude mice respectively. The transfection efficiency of LINC00242 was validated by qRT-PCR. **i–k** The effect of LINC00242 silencing on tumor volume (**i**), weight (**j**) and growth (**k**) were verified. N = 6; **P < 0.01 compared to lv-sh-NC group

To confirm whether LINC00242 modulates gastric cancer cell proliferation, apoptosis and glycolysis through miR-1-3p, the research transfected sh-LINC00242#1, sh-LINC00242#2 and/or miR-1-3p inhibitor into AGS and MGC-803 cells. Cells were separated into six groups: sh-NC + inhibitor NC group (cells transfected with empty plasmid), sh-LINC00242#1 group (cells transfected with shRNA-LINC00242#1), sh-LINC00242#2 group (cells transfected with shRNA-LINC00242#2), miR-1-3p inhibitor group (cells transfected with miR-1-3p inhibitor), Mix1 group (cells co-transfected with shRNA-LINC00242#1 + miR-1-3p inhibitor) and Mix2 group (cells co-transfected with shRNA-LINC00242#2 + miR-1-3p inhibitor). The transfection efficiency was validated by detecting LINC00242 and miR-1-3p expression in different experiment groups (Fig. 7a, b). LINC00242 knockdown suppressed cell viability and enhanced cell apoptosis in both MGC-803 and AGS, while inhibition of miR-1-3p revealed opposite impacts to LINC00242 on gastric cancer cell phenotype; inhibition of miR-1-3p markedly weakened the functions of LINC00242 silencing (Fig. 7c, d). As for the glycolysis course, silencing of LINC00242 restrained glucose consumption, lactate production, and ATP production; whereas inhibition of miR-1-3p revealed opposite impacts (Fig. 7e–g). In sum, sufficient evidences confirmed that LINC00242 modulated miR-9 expression, thus affecting gastric cancer cell proliferation and glycolysis progression in vitro.

In addition, the effect of LINC00242 on the growth and tumorigenic ability of gastric cancer in vivo was also measured in xenograft nude mice model. AGS cells pre-transfected with lentivirus-mediated shRNA-LINC00242#1, shRNA-LINC00242#2 or shRNA-control were subcutaneous injected into the back of nude mice respectively. The LINC00242 expression level in lv-sh-LINC00242#1 or lv-sh-LINC00242#2 group was notably decreased compared to lv-sh-NC group (*P* < 0.01, Fig. 7h). Knockdown of LINC00242 remarkedly suppressed tumor volume, weight and growth (*P* < 0.01, Fig. 7i–k). Therefore, LINC00242 downregulation attenuated tumorigenesis of gastric cancer in vivo.

## Discussion

Gastric cancer is an invasive malignant tumor with poor prognosis (Digklia and Wagner 2016). Tumorigenesis and tumor development partially depend on the reprogramming of tumor metabolism (Yuan et al. 2016). Warburg effect is a phenotypic characteristic of cancer metabolism. Targeting tumor metabolism, including aerobic glycolysis, may be a potential therapeutic strategy in directing treatment for gastric cancer (Vander Heiden and DeBerardinis 2017). In the current research, for the first time, we found that the *G6PD* is a key regulator of Warburg effect and paly the oncogenic role within gastric cancer. *G6PD* expression level was observably high-expressed in gastric cancer tissues and cells. Similar to our observation, Wang et al*.* identified *G6PD* is abnormally elevated and associated with poor clinical outcome in gastric cancer (Wang et al. 2012). Besides, a growing body of research manifested that *G6PD* participate in the crucial development progresses of multiple types of human tumor, including gastric cancer. Lu et al*.* proposed that overexpression of *G6PD* contributes to invasion and migration of hepatocellular carcinoma cells by inducing epithelial-mesenchymal transition (Lu et al. 2018). Zhang et al*.* reported that *G6PD* plays an activated role in colon cancer via promoting cell growth (Zhang et al. 2017). The biological function of *G6PD* to gastric cancer in the research was investigated in gastric cancer cells in vitro and xenograft mice in vivo. Knockdown of *G6PD* restrained gastric cancer cell proliferation and glycolysis in vitro and relieves the tumorigenesis of gastric cancer in vivo. All these outcomes uncovered that *G6PD* is closely related to the progression of gastric cancer by promoting the aerobic glycolysis.

A growing body of research manifested that quite a number of lncRNAs modulate protein-coding gene expression and involve in the crucial regulation processes of multiple types of human tumor, including in aerobic glycolysis (Liu et al. 2019a, b; Shankaraiah et al. 2018). For example, Xue et al*.* elucidated that lncRNA ABHD11-AS1 facilitates the proliferation and aerobic glycolysis of non-small-cell lung cancer (Xue et al. 2020). Wang et al*.* demonstrated that lncRNA LINRIS was high-expressed in colorectal cancer tissues from patients with poor overall survival (OS), and stabilizes IGF2BP2 and promotes the aerobic glycolysis in colorectal cancer (Wang et al. 2019a, b). The high LINC00174 expression represented a disadvantageous consequence in glioma patients and LINC00174 exacerbated glioma cell invasion, migration and glycolysis (Shi et al. 2019). In our study, the expression of lncRNA LINC00242 was memorably high-expressed in gastric cancer tissue samples and cells. Silencing of LINC00242 inhibited gastric cancer cell proliferation and glycolysis progression in vitro and attenuated tumorigenesis of gastric cancer in vivo. Similarly, previous studies revealed LINC00242 was highly expressed in gastric cancer tissues and cells and LINC00242 knockdown inhibited gastric cancer cell viability, migration and invasion, and tube formation of HBMVECs (Zhong et al. 2020). These outcomes announced that LINC00242 serves as an oncogene by facilitating the aerobic glycolysis in gastric cancer cells, therefore accelerating gastric cancer cell growth.

Recent studies elucidated that lncRNAs could serve as an endogenous miRNA sponges and participate in post-transcriptional regulation by interaction with miRNAs (Militello et al. 2017; Thomson and Dinger 2016). In consideration of miRNA also exerts a crucial role of aerobic glycolysis in gastric cancer, the present study screened that miR-1-3p could straight targeted bind to LINC00242 and *G6PD* 3′UTR, respectively. Anteriorly, miR-1-3p was known to restrain cell invasion and proliferation in gastric cancer by reducing STC2 (Ke et al. 2019). Besides, miR-1-3p sensitizes HGF-induced gefitinib-resistant human lung cancer cells through suppression of c-Met signaling and EMT (Jiao et al. 2018). Whereas, the influence of miR-1-3p on gastric cancer aerobic glycolysis still remains obscure. Hence, it was the first time to demonstrate that the overexpression of miR-1-3p inhibited the cell aerobic glycolysis and proliferation of gastric cancer cells. Notably, miR-1-3p expression was negatively corelative with LINC00242 and *G6PD* expression. Concerning the molecular mechanism, LINC00242 competitively regulated miR-1-3p, therefore offsetting miR-1-3p-mediated suppression on *G6PD*, indicating that LINC00242 modulates gastric cancer cell aerobic glycolysis and proliferation through miR-1-3p and *G6PD* signaling.

Nevertheless, limitations in this research are to be taken into consideration. Firstly, there could be other miRNAs sponged by LINC00242 or *G6PD* and involved in gastric cancer aerobic glycolysis, as well as other functional gene targets of miR-1-3p. Additionally, since all our experiments were carried out in mice and cells, the research outcomes might not be directly extrapolated to humans. For future researches, we may introduce tests on other animal models such as rats or rabbits.

## Conclusion

In summary, sufficient evidences were provided to feature the latent molecular mechanism of *G6PD* in gastric cancer aerobic glycolysis. We found that LINC00242 impose effects by sponging miR-1-3p expression, which negatively regulate *G6PD* expression, to affect gastric cancer cell proliferation and aerobic glycolysis. The finding of LINC00242/miR-1-3p/G6PD axis in gastric cancer facilitated us to better understand the development of gastric cancer, thus provided new therapeutic strategies for this fatal disease.

## Data Availability

Please contact the authors for data requests.

## Abbreviations

CCK-8: Cell counting Kit-8 kit
FBS: Fetal bovine serum
GC: Gastric cancer
GEO: Gene expression omnibus
*G6PD*: Glucose-6-phosphate dehydrogenase
GLUT1: Glucose transporter isoform 1
H&E: Hematoxylin and eosin
HK2: Hexokinase 2
IHC: Immunohistochemical
LDHA: Lactate dehydrogenase A
lncRNAs: Long non-coding RNAs
LINC00242: Long intergenic non-protein coding RNA 242
NCBI: National Center of Biotechnology Information
OS: Overall survival
PPP: Pentose phosphate pathway
